# Physiological and immunological responses of sea cucumber *Apostichopus japonicus* during desiccation and subsequent resubmersion

**DOI:** 10.7717/peerj.7427

**Published:** 2019-08-02

**Authors:** Shiying Hou, Zewei Jin, Wenwen Jiang, Liang Chi, Bin Xia, Jinghua Chen

**Affiliations:** 1 Marine Science and Engineering College, Qingdao Agricultural University, Qingdao, Shandong, China; 2 Weihai Ocean Vocational College, Weihai, Shandong, China; 3 College of Veterinary medicine, Qingdao Agricultural University, Qingdao, Shandong, China

**Keywords:** *Apostichopus japonicus*, Desiccation, Stress response, Oxidative damage, Non-specific immune, Antioxidant defense, Non-specific immunity

## Abstract

Desiccation is one of the extremely stressful situations experienced by aquatic animals, and sea cucumber usually suffers from desiccation stress during transportation without water. The present study was conducted to evaluate the effect of desiccation and subsequent resubmersion on physiological stress, oxidative damage, antioxidant status and non-specific immune response of *Apostichopus japonicus*, providing valuable information on the health management of sea cucumber culturing. Control and desiccation groups were set up, and each group has three replicates. After 1, 3 and 6 h of desiccation, individuals were resubmersed in aerated seawater for a 24 h recovery in three batches, which were represented as D1, D3 and D6, respectively. The results showed that glucose level in coelomic fluid of sea cucumber significantly decreased after desiccation, whereas lactate, cortisol and osmolality showed remarkable ascending trends. Thereafter, all stress parameters gently recovered towards normal levels as control group during 24 h resubmersion. The prolonged desiccation at D6 treatment induced the significant increases of malondialdehyde (MDA) and reactive oxygen species (ROS) contents, as well as relatively lower superoxide dismutase (SOD) and catalase (CAT) activities. During the period of desiccation and subsequent resubmersion, sea cucumber adjusted antioxidant defense to reduce the concentrations of MDA and ROS as a strategy for protecting against oxidative damage. Desiccation also had significant effects on non-specific immune parameters (total coelomocytes counts, TCC; complement C3; total nitric oxide synthase, T-NOS; lysozyme, LSZ; alkaline phosphatase, AKP) of *A. japonicus*, which could be recovered to some extent during resubmersion. In conclusion, less than 6 h of desiccation did not induce irreparable damage to sea cucumber, and was recommended for handling and shipping live sea cucumbers.

## Introduction

Sea cucumber *Apostichopus japonicus* (Selenka) is an important mariculture species in China. The farming scale has been rapidly expanded in the last decades due to the increasing market demand (Xia et al., 2017b). The production of this species has reached 220,000 t in 2017, with 110% increase compared to 2009 (MOAC, 2010; MOAC, 2018). In aquaculture practice, sea cucumbers usually suffer from desiccation during transportation without water. Desiccation is a common stressor experienced by aquatic animals (Lambert et al., 2018); however, desiccation tolerances are known to be discrepancy among species (Wells & Baldwin, 1995).

Previous studies have demonstrated that desiccation could cause serious metabolic and respiratory disturbances of organisms (Johnson & Uglow, 1985; Omori, Irawan & Kikutani, 1998; Haupt et al., 2006). Trushenski et al. (2010) found that desiccation significantly affected the physiological stress levels of cobia *Rachycentron canadum*, i.e., glucose, lactate, cortisol and osmolality, which were widely used as biomarkers under environmental stress (Pei et al., 2012; Xia et al., 2017a). Most reports were focused on oxidative stress and antioxidant response of aquatic animals (Romero et al., 2007; Xu et al., 2018). For example, Duan et al. (2016b) documented the changes of oxidative damage (reactive oxygen species, ROS; malondialdehyde, MDA; protein carbonyl, PC; lipid peroxidation, LPO) and antioxidant enzyme activities (superoxide dismutase, SOD; catalase, CAT; glutathione peroxidase, GPx; peroxidase, POD) in hepatopancreas of tiger shrimp *Penaeus monodon* after desiccation. However, there were few studies conducted on the effect of desiccation on immunologcial response of organisms, and it is crucial to understand the regulatory mechanism of resistance to desiccation stress (Cardinaud et al., 2014; Li et al., 2017). Meanwhile, aquatic animals could eliminate the metabolites in tissue and alleviate stress response after a period of submersion recovery, as reported by Liu et al. (2015) and Duan et al. (2016a).

Little is currently known about the effect of desiccation and subsequent resubmersion on physiological and immunological responses of sea cucumber. The objective of this study was to investigate the changes of physiological stress, oxidative damage, antioxidant status and non-specific immune response of *A. japonicus* during desiccation and subsequent resubmersion, providing valuable information on the health management of sea cucumber culturing.

## Materials and Methods

### Experimental design

The sea cucumbers with average wet weight of 20.08 ± 1.33 g were collected from a commercial farm in Qingdao, China, and immediately transported by cylinder aquaria (∼400 L capacity) with aerated seawater to the laboratory condition. All animals were acclimated for 3 weeks in circulating seawater system at 20 °C, salinity 30–32 PSU, dissolved oxygen above 6.5 mg L^−1^ and a 14 h light: 10 h dark photoperiod (Chen et al., 2018a). During the acclimation period, the sea cucumbers were fed with a formulated diet (fish meal, soybean meal and *Sargassum thunbergii* used as protein sources and squid liver oil as lipid source, containing 16.80% crude protein, 2.30% crude lipid and 10.23 kJ g^−1^ energy) and up to 5% of their total biomass per day. In order to avoid the changes in physiological status as a result of food intake, all animals were fasted for 3 days prior to the start of the experiment, and were also not fed throughout the period of desiccation and subsequent resubmersion (Trushenski et al., 2010; Lambert et al., 2018). After acclimation, the sea cucumbers were divided into two groups, i.e., control group and desiccation group. Each group contained 360 sea cucumbers that were randomly allocated into three cylinder aquaria as replicates, i.e., 120 individuals per aquarium. For the desiccation group, the sea cucumbers were performed in dissecting plates without water at air conditioning temperature of 20 °C indoor, and water-soaked gauze was used to maintain air humidity. After 1, 3 and 6 h of desiccation, 90 individuals were subsequently resubmersed in aerated seawater as same to acclimation condition for a 24 h recovery in three batches, which were represented as D1, D3 and D6, respectively. The control group was not exposed to any intentional experimental disturbance prior to sampling.

### Sample collection and determination

At the time points of 0, 1, 3 and 6 h post-desiccation and 3, 6, 12 and 24 h post-resubmersion, the coelomic fluid of five sea cucumbers were randomly sampled by a one mL disposable syringe and immediately mixed with an equal volume of anticoagulant. An aliquot of coelomic fluid was taken for determination of stress-related parameters, while the left separated coelomocytes that were resuspended in 600 μL cold 0.85% saline and then sonicated at 22 kHz for 25 s at 0 °C followed by centrifugation at 4,000× g for 10 min at 4 °C, to obtain the cells lysate supernatant for further immune-related assays (Chen et al., 2018b; Chen et al., 2018c). During the experiment, the survival rates at different time points of desiccation were calculated by the proportions of final living individuals to initial sea cucumbers.

Glucose in coelomic fluid was determined by Glucose Diagnostic Kits (Rsbio, China), lactate was analyzed enzymatically using Sigma Diagnostic Kits (Sigma, St. Louis, MO, USA) and cortisol was measured using Lodine [^125^I]-Cor RIA Kits (Jiuding Diagnostic, China) by radioimmunoassay following the manufacturer’s instructions (Pei et al., 2012). Osmolality was determined by a pressure osmometer (Fiske210; Advanced Instruments, Norwood, MA, USA). Glucose, lactate and cortisol levels in coelomic fluid were expressed as mmol L^−1^, while osmolality was expressed as mOsm kg^−1^.

The malondialdehyde (MDA) content and reactive oxygen species (ROS) production were also analyzed using commercial kits (Nanjing Jiancheng, China). MDA assay is based on measurement of the concentration of a pink chromogen compound that forms when MDA reacts with thiobarbituric acid and absorbs strongly at 532 nm (Wang et al., 2016). The production of ROS is assayed based on the fluorescent intensity of oxidant-sensitive probe dihydrorhodamine 123 (Xu et al., 2018). MDA and ROS contents in coelomic fluid were expressed as nmol L^−1^ and U mL^−1^, respectively. Superoxide dismutase (SOD) was determined by its ability to inhibit superoxide anion generated by xanthine and xanthine oxidase reaction system according to Ōyanagui (1984) with SOD Assay Kits (Nanjing Jiancheng, China). One SOD unit (U mL^−1^) was defined as the amount of enzyme required when inhibition rate reached 50% in a one mL reaction system. Catalase (CAT) was measured according to Góth (1989) using assay kits (Nanjing Jiancheng, China). One CAT unit (U mL^−1^) was defined as the amount catalyzing 1 μmol H_2_O_2_ per second.

Total coelomocytes were counted and calculated as cells mL^−1^ using a hemocytometer under light microscope at 400× magnification. Coelomocytes phagocytosis was determined by neutral red method (Zhao et al., 2016). The capability of coelomocytes phagocytosing neutral red was represented by the absorbance of 10^6^ cells. Complement C3 was measured according to Ma et al. (2017) with ELISA Kits (Nanjing Jiancheng, China). The concentration of complement C3 was expressed as μg mL^−1^. Total nitric oxide synthase (T-NOS) was determined by its catalytic ability to convert_*L*_-Arginine into NO according to Green et al. (1982) with T-NOS Assay Kits (Nanjing Jiancheng, China). One T-NOS unit (U mL^−1^) was defined as the amount of T-NOS producing 1 nmol NO per min. Lysozyme (LSZ) was assayed following the method of Yu et al. (2016) with a standard suspension of *Micrococcus luteus* cell walls that was ground with phosphate buffer solution (PBS) provided in the assay kits of Nanjing Jiancheng, China. One LSZ unit (U mL^−1^) was defined as the amount of enzyme required to decrease absorbance at a rate of 0.001 per min. Activity of alkaline phosphatase (AKP) was determined by the method of King (1965) using disodium phenyl phosphate as substrate with chemical detection kits (Nanjing Jiancheng, China). One AKP unit (U 100 mL^−1^) was defined as the amount of enzyme required to produce 1 μmol phenol. All enzymatic assays were conducted within 12 h after extraction.

### Statistical analysis

One-way analysis of variance (ANOVA) with Duncan’s test was used to compare the discrepancies in physiological and immunological parameters between the time points of desiccation and subsequent resubmersion. A probability level of 0.05 was used for rejection of the null hypothesis. Prior to analysis, raw data were diagnosed for normality of distribution and homogeneity of variance with Kolmogorov–Smirnov test and Levene’s test, respectively (Zar, 1999). All statistical analysis were performed with software SPSS for Windows release 16.0 (SPSS, Chicago, IL, USA).

## Results

### Survival rate

**Figure 1:**
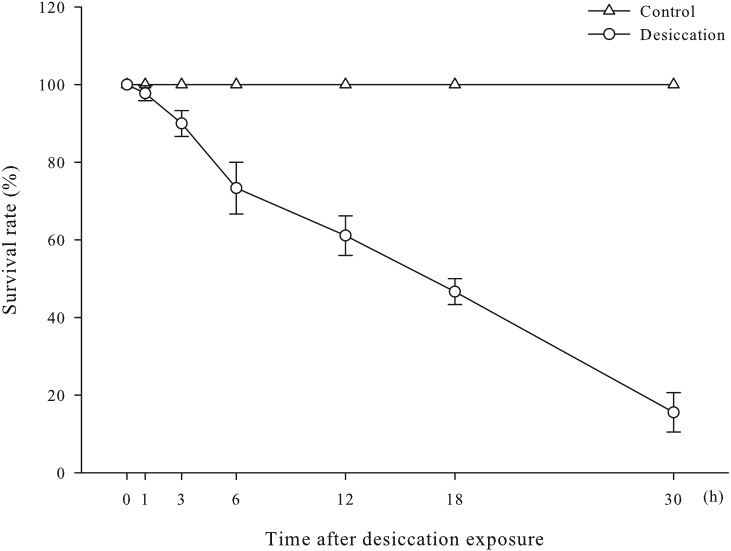
Survival rates of *A. japonicus* after desiccation. Data are mean ± SD.

Survival rates of *A. japonicus* after desiccation were present in Fig. 1. There was no significant differences in survival rate of sea cucumber at early stage of desiccation (0–1 h). After 1 h of desiccation, some individuals began to show the symptoms of head shook frequently, body distortion, evisceration and ulcerated skin, and successively died. As time prolonged, survival rate significantly declined and dropped to 15.56% at 30 h of desiccation (*p* < 0.05).

### Physiological stress

**Figure 2:**
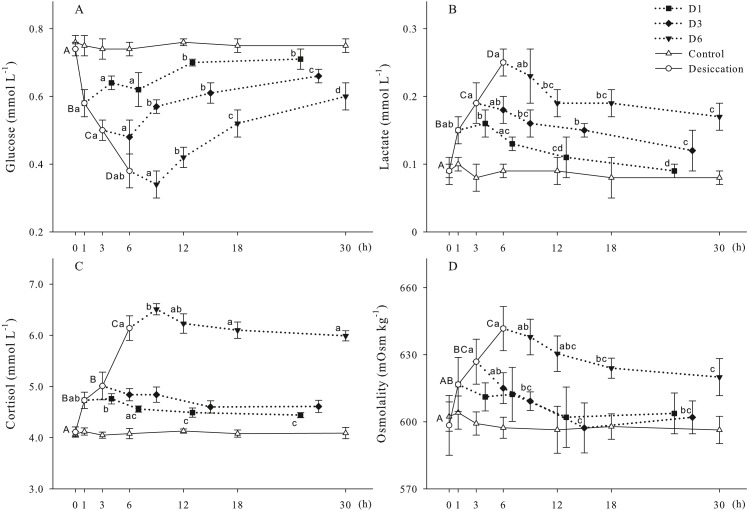
Glucose (A), lactate (B), cortisol (C) and osmolality (D) levels of *A. japonicus* during desiccation and subsequent resubmersion. Data are mean ± SD. Different superscript capital letters indicate significant differences between the time points of desiccation (*p* < 0.05), while different lowercase letters indicate significant differences between the time points of resubmersion (*p* < 0.05).

The present study investigated the physiological stress levels of *A. japonicus* during desiccation and subsequent resubmersion (Fig. 2). After desiccation, glucose level in coelomic fluid significantly decreased as time prolonged and dropped to the lowest values of 0.38 ± 0.05 mmol L^−1^ at 6 h of desiccation. During the resubmersion period, glucose at D1 treatment had significant differences between the sampling time points (*p* < 0.05), and gradually returned to normal levels as control group after a 24 h recovery. Although a temporal decline in glucose levels of D3 and D6 treatments was observed at 3 h of resubmersion, both then showed ascending trends during 3-24 h recovery. For all treatments, glucose levels at 12 and 24 h of resubmersion were significantly higher than those of 0 and 3 h (*p* < 0.05). Conversely, lactate level in coelomic fluid elevated significantly after desiccation and showed remarkable descending trends during resubmersion (*p* < 0.05). D3 and D6 groups at 24 h of resubmersion had significant higher lactate levels compared to control group (*p* < 0.05). Lactate levels at 12 and 24 h recovery were significantly lower than those of 0 h (*p* < 0.05).

As shown in Fig. 2, cortisol level in coelomic fluid of sea cucumber significantly increased as time prolonged and was highest at 6 h of desiccation (*p* < 0.05). During the resubmersion period, cortisol level at D1 treatment significantly decreased (*p* < 0.05). Cortisol at D6 treatment did not peak until 3 h post-desiccation and remained elevated for hours after stress challenge (*p* < 0.05). Although no significant differences was observed between the time points of resubmersion, cortisol level at D3 treatment showed a slowly downward trend. There were significantly differences in osmolality of sea cucumberbetween the time points of resubmersion at D3 and D6 groups (*p* < 0.05), however, no significant differences was observed at D1 treatment (*p* > 0.05).

### Oxidative damage and antioxidant status

**Figure 3:**
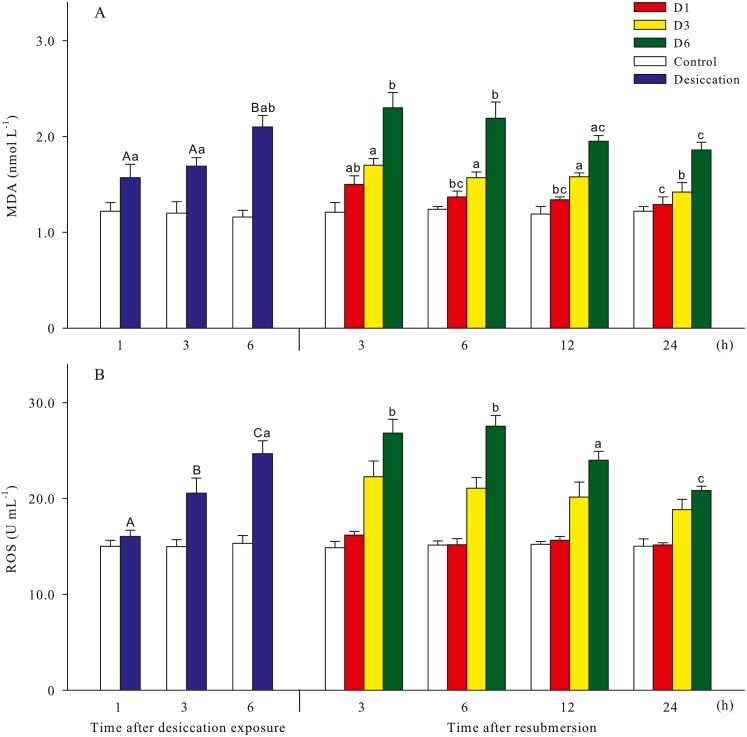
Oxidative damage parameters (A: malondialdehyde, MDA; B: Reactive oxygen species, ROS) of *A. japonicus* during desiccation and subsequent resubmersion. Data are mean ± SD. Different superscript capital letters indicate significant differences between the time points of desiccation (*p* < 0.05), while different lowercase letters indicate significant differences between the time points of resubmersion (*p* < 0.05).

**Figure 4:**
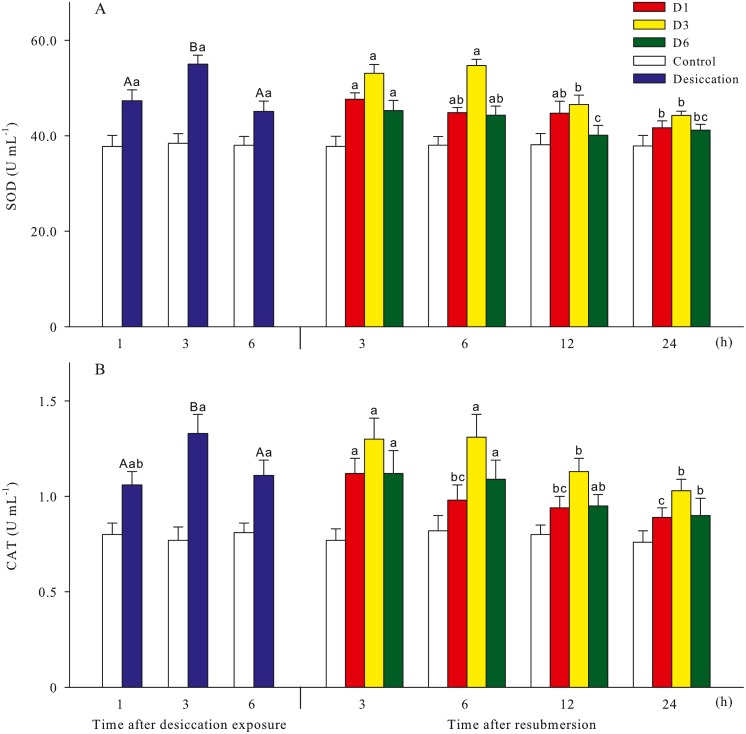
Antioxidant enzyme activities (A: superoxide dismutase, SOD; B: catalase, CAT) of *A. japonicus* during desiccation and subsequent resubmersion. Data are mean ± SD. Different superscript capital letters indicate significant differences between the time points of desiccation (*p* < 0.05), while different lowercase letters indicate significant differences between the time points of resubmersion (*p* < 0.05).

Malondialdehyde (MDA) and reactive oxygen species (ROS) contents of *A. japonicus* during desiccation and subsequent resubmersion were shown in Fig. 3. In the present study, ROS in coelomic fluid of sea cucumber significantly increased as time prolonged after desiccation (*p* < 0.05). Meanwhile, MDA content at 6 h of desiccation were significantly higher than those of 1 h and 3 h (*p* < 0.05). For all treatments, MDA showed obvious descending trends during resubmersion (*p* < 0.05). ROS content at D6 group firstly increased and then significantly decreased (*p* < 0.05); however, no significant differences was observed between the time points of resubmersion at D1 and D3 groups (*p* > 0.05). In Fig. 4, SOD and CAT activities of sea cucumebr significantly increased and then declined after desiccation (*p* < 0.05). During the resubmersion period, SOD and CAT activities at D1, D3 and D6 treatments all significantly deceased as time prolonged (*p* < 0.05).

### Non-specific immune response

**Figure 5:**
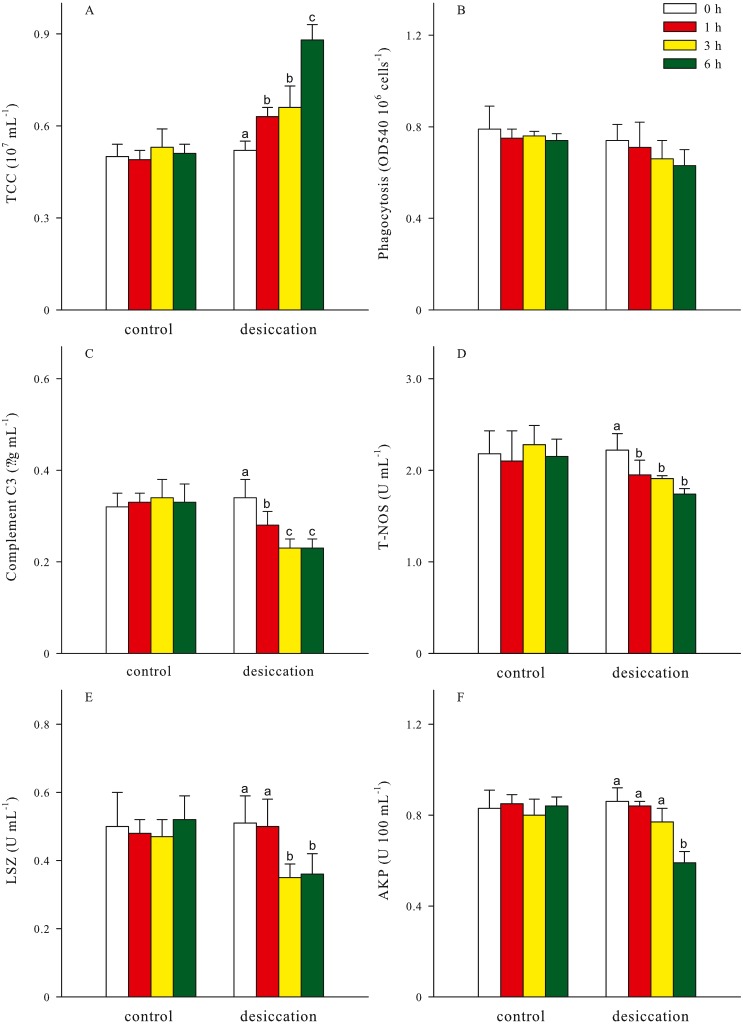
Non-specific immune response (A: total coelomocytes counts, TCC; B: phagocytosis; C: complement C3; D: total nitric oxide synthase, T-NOS; E: lysozyme, LSZ; F: alkaline phosphatase, AKP) of *A. japonicus* after desiccation exposure. Data are mean ± SD. Different lowercase letters indicate significant differences between the time points of desiccation (*p* < 0.05).

Total coelomocytes counts (TCC), phagocytosis, complement C3, total nitric oxide synthase (T-NOS), lysozyme (LSZ), alkaline phosphatase (AKP) of *A. japonicus* after desiccation were present in Fig. 5. In the present study, TCC in coelomic fluid significantly increased as time prolonged after desiccation, with highest values of 0.88 ± 0.05 ×10^7^ mL^−1^ at D6 treatment (*p* < 0.05). Phagocytosis showed a downward trend after desiccation despite no significant differences was observed between the time points (*p* > 0.05). During the desiccation period, complement C3 content, T-NOS, LSZ and AKP activities all significantly decreased as time prolonged (*p* < 0.05, Fig. 5).

For all treatments, TCC in coelomic fluid of sea cucumber exhibited remarkable declining trends after resubmersion, and the lowest values were observed at 24 h of resubmersion (*p* < 0.05, Table 1). There was no significant differences in phagocytosis between the time points of resubmersion (*p* > 0.05). Other immune-related parameters including complement C3, T-NOS, LSZ and AKP at D3 and D6 treatments were significantly affected during resubmersion and recovered towards normal levels as control group (*p* < 0.05). However, no significant differences between the time points of resubmersion was observed at D1 treatment (*p* > 0.05). Both of complement C3 and T-NOS showed increasing trends after resubmersion with corresponding to the significantly lower values at 1 h of desiccation in Fig. 5.

**Table 1:** Non-specific immune response (total coelomocytes counts, TCC; phagocytosis; complement C3; total nitric oxide synthase, T-NOS; lysozyme, LSZ; alkaline phosphatase, AKP) of *A. japonicus* at different treatments after resubmersion. Data are mean ± SD. Different lowercase letters indicate significant differences between the time points of resubmersion (*p* < 0.05).

Groups	Time after resubmersion (h)	TCC (10^7^ mL^−1^)	Phagocytosis (OD540 10^6^ cells^−1^)	Complement C3 (μg mL^−1^)	T-NOS (U mL^−1^)	LSZ (U mL^−1^)	AKP (U 100 mL^−1^)
D1	0	0.63 ±0.03^a^	0.71 ± 0.11	0.28 ± 0.03	1.95 ± 0.16	0.50 ± 0.08	0.84 ± 0.02
	3	0.65 ±0.05^a^	0.69 ± 0.10	0.28 ± 0.02	1.95 ± 0.06	0.48 ± 0.04	0.80 ± 0.04
	6	0.58 ±0.03^a^	0.71 ± 0.06	0.27 ± 0.05	2.05 ± 0.04	0.54 ± 0.06	0.83 ± 0.06
	12	0.51 ±0.03^b^	0.74 ± 0.06	0.31 ± 0.02	1.98 ± 0.07	0.49 ± 0.06	0.83 ± 0.04
	24	0.50 ±0.03^b^	0.77 ± 0.04	0.32 ± 0.03	2.10 ± 0.10	0.51 ± 0.05	0.81 ± 0.03
D3	0	0.66 ±0.07^a^	0.66 ± 0.08	0.23 ±0.02^ab^	1.91 ±0.03^a^	0.35 ±0.04^a^	0.77 ±0.06^a^
	3	0.64 ±0.03^a^	0.66 ± 0.07	0.21 ±0.02^a^	1.93 ±0.09^a^	0.40 ±0.03^ab^	0.77 ±0.02^a^
	6	0.62 ±0.03^a^	0.62 ± 0.07	0.24 ±0.02^abc^	1.88 ±0.07^a^	0.42 ±0.05^b^	0.81 ±0.03^ab^
	12	0.52 ±0.04^b^	0.70 ± 0.04	0.26 ±0.02^bc^	1.98 ±0.06^ab^	0.45 ±0.02^bc^	0.86 ±0.02^b^
	24	0.50 ±0.06^b^	0.67 ± 0.07	0.27 ±0.04^c^	2.11 ±0.10^b^	0.49 ±0.02^c^	0.87 ±0.02^b^
D6	0	0.88 ±0.05^a^	0.63 ± 0.07	0.23 ±0.02^ab^	1.74 ±0.06^ab^	0.36 ±0.06^ab^	0.59 ±0.05^a^
	3	0.84 ±0.04^a^	0.62 ± 0.04	0.20 ±0.02^a^	1.70 ±0.10^a^	0.33 ±0.04^a^	0.56 ±0.06^a^
	6	0.86 ±0.06^a^	0.60 ± 0.08	0.19 ±0.03^a^	1.67 ±0.05^a^	0.33 ±0.04^a^	0.55 ±0.04^a^
	12	0.80 ±0.03^ab^	0.64 ± 0.08	0.23 ±0.02^ab^	1.85 ±0.04^b^	0.42 ±0.03^bc^	0.70 ±0.06^b^
	24	0.73 ±0.03^b^	0.70 ± 0.07	0.25 ±0.02^b^	1.82 ±0.05^b^	0.48 ±0.03^c^	0.71 ±0.07^b^

## Discussion

Desiccation has been proven to induce adverse effects on survival of aquatic animals (Xu et al., 2018). Duan et al. (2016a) found obvious stress symptoms in hepatopancreas and significantly decreased the survival rates of kuruma shrimp *Marsupenaeus japonicus* after 3 h of desiccation. It has been demonstrated that desiccation tolerance was related to species, size and health status of aquatic animals (Wells & Baldwin, 1995; Cardinaud et al., 2014).

Glucose is an essential energy substance for animal metabolism and lactate is an intermediated production of energy metabolism (Barnett & Pankhurst, 1998), which are known to be biomarkers of physiological stress in sea cucumber (Pei et al., 2012; Chen et al., 2018b). In the present study, significantly elevated lactate level and declined glucose level in coelomic fluid of sea cucumber implied high energy consumption after desiccation. During the resubmersion period, glucose and lactate gradually recovered towards normal levels as control group. Previous studies have demonstrated that organisms could eliminate the metabolites in tissue and alleviate stress response after a period of recovery (Oliveira, Rossi & Da Silva, 2001; Duan et al., 2016a). Lambert et al. (2018) also found that whereas the reduction in plasma glucose content of Atlantic stingray *Hypanus sabinus* following air exposure gently recovered to a relatively higher level after recovery in water, the increase in blood lactate concentration was slower to dissipate.

Plasma cortisol in many aquatic animals increased under environment stress (Ruane, Carballo & Komen, 2002; Thomas et al., 2007). Elevated cortisol is also associated with the increasing lactate level (Mommsen, Vijayan & Moon, 1999), as reported by our study. Trushenski et al. (2010) found that cortisol was cleared quickly from the bloodstream of cobia *Rachycentron canadum* within 2 h post-challenges of low water and air exposure, and similar rapid and brief cortisol responses were also reported by other studies (Lima et al., 2006; Di Marco et al., 2008; Davis & McEntire, 2009). However, cortisol in coelomic fluid of sea cucumber did not return to normal levels as control group within 24 h recovery, especially for D6 treatment, which could be attribute to a longer lasting metabolic response induced by overlong desiccation. Previous studies have documented that osmolyte homeostasis in aquatic animals could be disturbed by various environmental stress (Hoffmayer, Hendon & Parson, 2012). Temporary osmoregulatory dysfunction is a common secondary effect of desiccation stress (Cicia et al., 2012). In the present study, osmolality in coelomic fluid quickly increased after desiccation, and then slowly returned towards normal levels during resubmersion. The effect of desiccation on osmolality of sea cucumber was in accordance with the commonly observed osmotic fluxes in other stressed fishes (Trushenski et al., 2010). This prolonged imbalance of cortisol and osmolality at D6 treatment demonstrated that desiccation has long-term negative effects, maybe requiring extensive recovery (Lambert et al., 2018).

ROS are chemically reactive species containing oxygen, such as superoxide (${O}_{2}^{-}$), hydrogen peroxide (*H*_2_*O*_2_), hydroxyl free radical (OH^−^) and single oxygen (^1^O_2_) (Hayyan, Hashim & AINashef, 2017). In a biological context, ROS are formed as a natural byproduct of oxygen metabolism and play important roles in cell signaling and homeostasis (Yu, 1994). However, as highly reactive molecules, ROS could increase dramatically and lead to oxidative stress when an imbalance occurs between producing and removing ROS under environmental stress (Huo et al., 2018). Previous studies have demonstrated that environmental stress could induce the excessive production or accumulation of ROS in aquatic animals, e.g., fish (Pérez-Jiménez et al., 2012; Wang et al., 2018), crustaceans (Liang et al., 2016; Han et al., 2018) and sea cucumber (Qi et al., 2016; Wang et al., 2016), causing serious tissue damage and resulting in various disease outbreaks. MDA is the terminal product of lipid peroxidation by ROS, which could reflect stress by oxyradical in organisms and considered as an important biomarker of oxidative damage to cell membrane (Moore & Roberts, 1998; Del Rio, Stewart & Pellegrini, 2005). As reported by Liu et al. (2015) and Xu et al. (2018), desiccation could significantly increase the MDA content of shrimp. Our results also showed that the prolonged desiccation at D6 treatment induced the sustainable formation of ROS and lipid peroxidation in membranes, which led to significantly higher MDA contents in coelomic fluid of sea cucumber.

In order to protect against the deleterious effects of ROS, organisms have developed a complex antioxidant system consisting of enzymatic and non-enzymatic detoxification mechanisms, to counteract oxidative stress and prevent oxidative damage (Afonso et al., 2007; Duan et al., 2016b). Antioxidant enzymes such as superoxide dismutase (SOD) and catalase (CAT) are known to be involved in environmental stress response of *A. japonicus* (Jiang et al., 2018; Zhang et al., 2018). SODs are a class of enzymes that catalyze the dismutation of O_2_ − into H_2_O_2_ and molecular oxygen, and then H_2_O_2_ is transformed to water and oxygen by CAT (Chen et al., 2018c). The relatively lower SOD and CAT activities might account for the highest ROS and MDA contents at 6 h of desiccation. Previous studies have demonstrated that the prolonged exposure to stress could reduce the activities of antioxidant enzymes (Wang et al., 2011; Xu et al., 2018). However, oxidative damage and antioxidant responses by desiccation stress did not cause irreversible alteration to sea cucumber. Duan et al. (2016a) also confirmed that antioxidant enzyme activities could return to normal levels after the kuruma shrimp *Marsupenaeus japonicus* was resubmersed in seawater. MDA and ROS also showed obvious downward trends, which demonstrated that sea cucumber could adjust antioxidant defence to reduce the concentrations of ROS and MDA as a strategy for protecting against oxidative damage (Romero et al., 2007).

It is commonly believed that immune response of sea cucumber is typical invertebrates’ non-specific immune, depending on immune defence reaction of body cavity cells and numerous humoral defence factors (Cooper, Uhlenbruck & Valembois, 1992; Li et al., 2016). The coelomocytes play an important role in immune system of sea cucumber, which can engulf and/or encapsulate foreign antigens and express variable effector mechanisms (Chia & Xing, 1996). Holm et al. (2008) found that the increased coelomocytes numbers observed in response to lipopolysaccharide and concanavalin A were reflected in an induced cell proliferation in coelomic epithelium of sea star *Asterias rubens*. Thus, we speculated that desiccation could induce cell proliferation in coelomic epithelium and resulted in the increment of coelomocytes. Or it attributed to the epidermal waterloss from coelomic fluid of sea cucumber during desiccation (Tan et al., 2017). Phagocytosis of coelomocytes was the primary line of immune defence in sea cucumber, and widely used to evaluate its defence ability against pathogens (Xing, Leung & Chia, 1998). Desiccation also induced the fall of respiratory burst and the inhibition of hemocyte morphological activation of mussel *Mytilus galloprovincialis*, suggesting a potential depression of the phagocytosis process (Mosca et al., 2013). In addition, the declining proportion of phagocytes in coelomic fluid of sea cucumber might account for the depression of phagocytosis under desiccation stress (Oweson et al., 2010).

Complement components can bind formulate the membrane attack complex with pathogens and lead to cell lysis (Boshra, Li & Sunyer, 2006). The relatively lower complement C3 at 3 and 6 h of desiccation could disrupt the innate immune system of sea cucumber resisting the invasion of pathogens. NOS is responsible for the production of NO, which is considered to be important mediator in reducing oxidative stress and enhancing immune response system (Zhao et al., 2012). T-NOS activities at 1–6 h of desiccation were significantly lower than control group, also suggesting the depression of non-specific immune response induced by desiccation stress. As an important hydrolytic enzyme, LSZ can eliminate bacteria through hydrolysis of peptidoglycan in cell wall (Callewaert & Michiels, 2010), and it commonly exists in coelomocytes and coelomic fluid of sea cucumber (Yu et al., 2016; Chen et al., 2018c). Previous studies have demonstrated that LSZ would correspondingly change as environmental factors beyond the optimum conditions of organisms (Huo et al., 2018). In the present study, the decrement of LSZ activities implied a low immunocompetence at 3–6 h of desiccation. AKP activity in coelomic fluid, as a reliable index in the assessment of immune status, was also down-regulated at 6 h of desiccation (Zhang et al., 2018). Desiccation influenced the non-specific immune responses of *A. japonicus*, but did not cause irreparable damages to sea cucumber. This study further proved that the tolerance to desiccation and time needed to recover to basal metabolism are species-specific characteristics (Romero et al., 2007; Xu et al., 2018).

## Conclusion

The present study demonstrated that desiccation could cause physiological stress and oxidative damage of *A. japonicus*, and influenced antioxidant status and non-specific immune response of sea cucumber. However, desiccation stress did not induce irreparable damage to sea cucumber within 6 h exposure. Glucose, lactate, cortisol and osmolality gradually recovered towards normal levels as control group after resubmersion, although 6 h of desiccation had long-term negative effects. During desiccation and subsequent resubmersion, sea cucumber adjusted antioxidant defence to reduce the concentrations of ROS and MDA as a strategy for protecting against oxidative damage. Meanwhile, most of immunological parameters were significantly affected by desiccation stress and could be recovered to some extent during 24 h resubmersion. In consideration of survival rate and various indicators, less than 6 h of desiccation was recommended and a recovery resubmersion as long as possible would be helpful for handling and shipping live sea cucumbers.

##  Supplemental Information

10.7717/peerj.7427/supp-1 Supplemental Information 1 Raw data for suvival rate of sea cucumber after disiccation exposure

10.7717/peerj.7427/supp-2 Supplemental Information 2 Raw data for physiological stress levels of sea cucumber during desiccation and subsequent resubmersion

10.7717/peerj.7427/supp-3 Supplemental Information 3 Raw data for oxidative damage parameters of sea cucumber during desiccation and subsequent resubmersion

10.7717/peerj.7427/supp-4 Supplemental Information 4 Raw data for antioxidant enzyme activities of sea cucumber during desiccation and subsequent resubmersion

10.7717/peerj.7427/supp-5 Supplemental Information 5 Raw data for non-specific immune response of sea cucumber duirng desiccation and subsequent resubmersion

## Additional Information and Declarations

### Data Availability

The following information was supplied regarding data availability: The raw data are available in the Supplemental Files. TAD, time after desiccation; TARS1, TARS3 and TARS6 represent time after recovery submersion of 1, 3, 6 h desiccation exposure, respectively.
